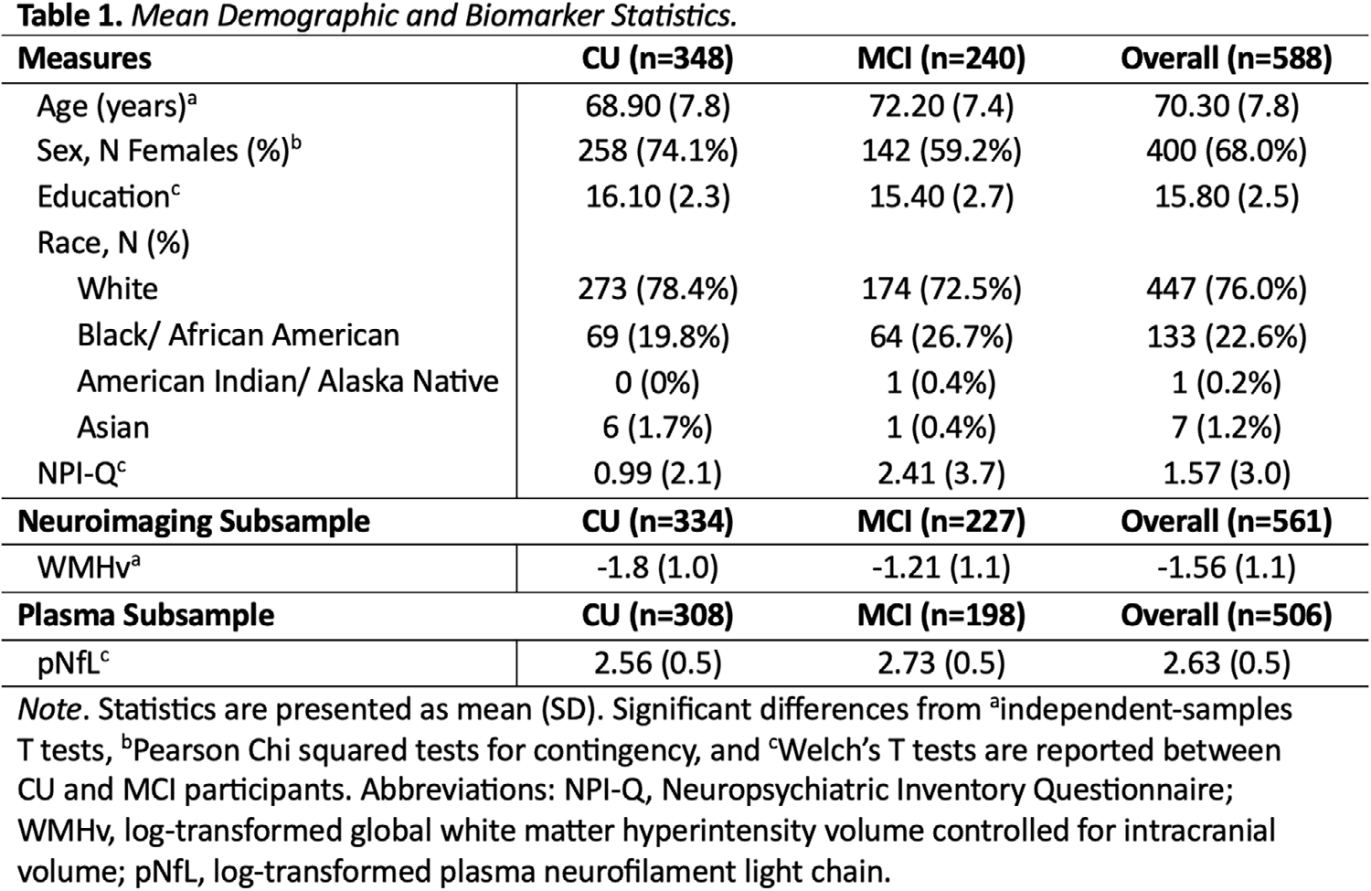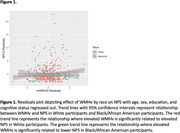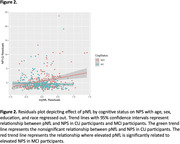# The Relationship of Neuropsychiatric Symptoms and ADRD Pathologies

**DOI:** 10.1002/alz.093933

**Published:** 2025-01-09

**Authors:** Julia R. Bacci, Marc D. Rudolph, Michelle M. Mielke, Suzanne Craft, James R. Bateman, Samuel N. Lockhart

**Affiliations:** ^1^ Wake Forest University School of Medicine, Winston‐Salem, NC USA

## Abstract

**Background:**

Neuropsychiatric symptoms (NPS) in the Alzheimer’s Disease (AD) clinical spectrum are common, yet the pathological underpinnings are unclear. Prior research has been inconsistent, attributed in part to concomitant co‐pathologies. Additionally, prior modeling approaches have disregarded the distributional properties of right‐skewed NPS data and utilized predominantly racially homogenous cohorts. Thus, we examined the relationship between NPS and markers of vascular injury and neurodegeneration using generalized linear models (GLM) in a diverse community‐based sample. Of additional interest was whether these relationships differed by cognitive status or race.

**Methods:**

We evaluated cross‐sectional relationships of brain white matter hyperintensity volume (WMHv) and plasma neurofilament light chain (pNfL) to NPS (NPI‐Q total severity scores) in 588 Wake Forest ADRC Clinical Core study participants. Analyses included participants with consensus diagnoses of cognitively unimpaired (CU; N = 348) and mild cognitive impairment (MCI; N = 240). WMHv and pNfL were log‐transformed prior to model entry. GLMs included age, sex, education, race, and cognitive status as covariates and pathologies as predictor variables. Secondary models included aforementioned covariates and pathology by (a) cognitive status and (b) race interaction terms to investigate how these associations differed.

**Results:**

WMHv was not significantly associated with NPS (N = 561; ß = 0.203; p = 0.053); further, we did not observe an interaction between cognitive status and WMHv (N = 561; ß = ‐0.055; p = 0.800). However, a significant race by WMHv interaction on NPI‐Q was demonstrated (N = 561; ß = ‐0.414; p = 0.016). In White participants, elevated WMHv was significantly related to elevated NPS (N = 434; ß = 0.325; p = 0.006); whereas in Black/African American participants elevated WMHv was significantly related to lower NPS (N = 119; ß = ‐0.239; p = 0.049). pNfL was not significantly associated with NPS (N = 506; ß = 0.149; p = 0.576). A significant pNfL by cognitive status interaction on NPI‐Q was found (N = 506; ß = ‐1.055; p = 0.029; Figure 2), where elevated pNfL was related to elevated NPS in MCI participants (N = 198; ß = 1.656; p = 0.004) but not CU participants (N = 308; ß = ‐0.316; p = 0.260). However, we did not observe an interaction between race and pNfL (N = 506; ß = 0.185; p = 0.678).

**Conclusion:**

Consistent with previous work we demonstrate associations of pNfL and WMHv with NPS. Future analyses with this cohort will examine additional biomarkers, including amyloid‐beta and tau measured in CSF, plasma, and PET.